# An Electrochemical System for Gaseous ClO_2_ Generation Using TiO_2_ Nanorod Array Cathodes Toward Fruit Preservation

**DOI:** 10.3390/ma19091674

**Published:** 2026-04-22

**Authors:** Luyi Pang, Junyuan Jiang, Rengui Guan, Yanyang Han, Shanshan Liu, Shasha Jiang, Wei Cui, Tao He

**Affiliations:** College of Chemistry and Chemical Engineering, Yantai University, Yantai 264005, China; ply20001203@s.ytu.edu.cn (L.P.); jjunyuan@s.ytu.edu.cn (J.J.); guanrengui@ytu.edu.cn (R.G.); yyhan@ytu.edu.cn (Y.H.); liushanshan@ytu.edu.cn (S.L.); jiangss0903@ytu.edu.cn (S.J.); ytuht@ytu.edu.cn (T.H.)

**Keywords:** electrocatalysis, ClO_2_ generation, TiO_2_ nanorod arrays, fruit preservation

## Abstract

The efficient on-demand generation of ClO_2_ is critical for disinfection and food preservation. However, the development of safe and efficient strategies for gaseous ClO_2_ production remains challenging. Herein, we report a stable and efficient electrochemical system for ClO_2_ production based on rutile TiO_2_ nanorod arrays (TiO_2_ NAs). Electrochemical optimization suggests that a cathodic potential of −0.10 V (vs. Ag/AgCl) in an electrolyte solution of 1 M NaClO_3_ with 5 M H_2_SO_4_ achieves the highest ClO_2_ production efficiency. Mechanistic studies reveal that ClO_2_ generation proceeds via an O_2_-induced pathway, in which electrochemically generated H_2_O_2_ from 2-e^−^ O_2_ reduction reacts in situ with ClO_3_^−^ to form ClO_2_, eliminating the need for external H_2_O_2_ storage and significantly improving operational safety. Furthermore, when decorated with RuO_x_ nanoparticles, TiO_2_ NA cathodes achieve enhanced catalytic performance and excellent stability. In addition, the generated ClO_2_ in the electrolyte solution can be delivered via gas pumping. This ClO_2_ atmosphere exhibits antibacterial efficiencies exceeding 99% against *Escherichia coli* and *Staphylococcus aureus*, and significantly reduced weight loss and preserved fruit hardness in longan samples during 8 days of storage. Overall, this work presents a safe, efficient approach for ClO_2_ generation with strong potential for practical disinfection in the food preservation field.

## 1. Introduction

Microbial contamination is a major challenge in food preservation, threatening shelf life, quality, and safety across a wide range of food products, leading to food spoilage and posing serious risks to food safety and public health. Disinfection has always been an essential hygienic measure in food preservation to control the growth of microorganisms such as bacteria, fungi and mold [1,2]. At present, a variety of chemical disinfectants have been widely applied in food preservation and postharvest sanitation, including organic acids (e.g., benzoate [3] and propionate [4]), chlorine-based compounds (Cl_2_ and hypochlorites) [5,6], SO_2_ [7], nitrites [8], O_3_ [9] and so on. All of these agents are effective in suppressing microbial growth; however, each of them suffers from intrinsic limitations that restrict their practical application. For example, organic acids, acting as either antimicrobials or antioxidants, typically require relatively high concentrations to achieve effectiveness and may affect sensory quality of food [10]. SO_2_ fumigation, while effective for certain fruits, suffers regulatory restrictions due to its intrinsic toxicity [11]. O_3_, a powerful oxidant, suffers poor stability and low water solubility, thus requiring on-site production with relatively high operational costs [12,13].

In this context, ClO_2_ has emerged as one of the most promising disinfectants. It features strong oxidizing power, broad-spectrum antimicrobial activity, rapid sterilization, and prolonged inhibitory effects. Furthermore, unlike molecular Cl_2_, ClO_2_ prevents the formation of harmful organochlorines, making it an environmentally friendly oxidant highly recommended by the World Health Organization (WHO) [14,15]. Notably, ClO_2_ can be applied in gaseous form, enabling effective penetration and elimination of microorganisms even at low concentrations. In addition, ClO_2_ readily decomposes into Cl^−^ ions and O_2_, which reduces the risk of toxic residue accumulation on food surfaces. All of these makes ClO_2_ fumigation particularly advantageous for delicate fruits.

Despite the above advantages, the practical application of gaseous ClO_2_ remains limited since ClO_2_ products are largely dependent on aqueous solutions. Although aqueous ClO_2_ generation systems can deliver gaseous ClO_2_ via gas stripping, they typically rely on the use and storage of hazardous reactants such as H_2_O_2_, which brings about additional safety concerns [16,17,18,19]. An alternative approach is based on slow-release systems, such as ClO_2_ gels or capsules, typically utilizing porous carriers to regulate the ClO_2_ release triggered by environmental humidity or pH change [20,21,22]. However, their release kinetics are highly dependent on environmental conditions and are difficult to precisely control, leading to non-uniform ClO_2_ distribution and potential overexposure. Therefore, a safe, efficient, and controllable gaseous ClO_2_ generation system is highly desirable.

Accordingly, electrochemical ClO_2_ generation has attracted significant attention and has been extensively investigated, owing to its precise control and ease of operation. Various electrochemical systems have been developed, focusing on different electrocatalytic reactions or designs for electrolysis cells. For example, Pillai and co-workers reported the use of a RuO_2_ anode for electrochemical generation of dissolved ClO_2_ in an undivided electrochemical cell, demonstrating of the feasibility of noble-metal-based catalytic oxidation pathways [23]. Wu et al. used NaClO_3_-containing brine as the electrolyte to produce ClO_2_ via anodic oxidation, highlighting the importance of electrolyte composition in regulating ClO_2_ formation efficiency [24]. The Rodrigo group systematically investigated the effects of various operation factors on the electrochemical generation of H_2_O_2_ and ClO_3_^−^ (reactants for chemical formation of ClO_2_), including temperature, electrolyte composition, fluid dynamics and so on [25,26]. These studies collectively demonstrate substantial progress in understanding dissolved-phase ClO_2_ generation and its governing electrochemical parameters. However, most reported systems are still limited to dissolved ClO_2_ production using conventional electrode configurations, and the discussion is largely confined to mechanistic or efficiency-oriented studies rather than realistic application-driven scenarios. In particular, practical applications such as gaseous ClO_2_-based antibacterial disinfection, food preservation, or postharvest freshness maintenance remain rarely addressed. Therefore, there is still a critical need to develop low-cost and efficient electrode systems capable of controllable gaseous ClO_2_ generation for application-relevant scenarios.

Herein, we report a gaseous ClO_2_ generation system based on TiO_2_ nanorod arrays. TiO_2_ nanorod arrays were selected as the electrode platform due to their high structural stability, excellent corrosion resistance in strong acidic media, and efficient directional electron transport capability [27,28]. The vertically aligned rutile TiO_2_ cathode showed effective catalytic activity toward ClO_2_ production in H_2_SO_4_/NaClO_3_ electrolyte via cathodic electrolysis, while RuO_x_ decoration enhanced catalytic activity and exhibited excellent stability under acidic conditions. In addition, our system enables in situ generation of H_2_O_2_ via O_2_ reduction, avoiding the use and storage of external H_2_O_2_. When ClO_2_ generated in the electrolyte solution was delivered into a gaseous environment, effective and sustained antimicrobial activity against *E. coli* and *S. aureus* was achieved. Moreover, the system demonstrates practical applicability for fruit preservation, as evidenced by significantly reduced microbial spoilage and improved maintenance for longan during 8-day storage. This study has established a promising prototype for safe, efficient, and scalable electrochemical ClO_2_ generation, highlighting its potential for practical sterilization and food preservation applications.

## 2. Materials and Methods

### 2.1. Materials

Sodium chlorate (NaClO_3_), hydrochloric acid and sulfuric acid were purchased from Sinopharm Chemical Reagent Co., Ltd. (Shanghai, China); tetrabutyl titanate was purchased from Alfa Aesar Chemical Co., Ltd. (Tianjin, China); *Escherichia coli* (*E. coli*, ATCC25922) and *Staphylococcus aureus* (*S. aureus*, ATCC 25923) were obtained from ATCC. Peptone was purchased from Beijing AOBOX Bio-technology Co., Ltd. (Beijing, China); Beef extract powder was purchased from Shanghai Boer Chemical Reagents Co., Ltd. (Shanghai, China)

### 2.2. Synthesis of TiO_2_ Nanorod Arrays (TiO_2_-NAs)

Slides of fluorine-doped tin oxide (FTO) glass (6 × 2 cm^2^) were ultrasonically cleaned with acetone, ethanol, and deionized water, and then dried under N_2_ for use as current collectors. To grow the TiO_2_ NAs, a precursor solution was first prepared by mixing 10 mL of hydrochloric acid and 10 mL of deionized (DI) water, followed by the dropwise addition of 0.535 mL tetrabutyl titanate under stirring. The solution and freshly cleaned FTO slides (conductive side facing down) were sealed in a Teflon-lined stainless-steel autoclave and heated at 150 °C for 12 h. After cooling to room temperature, the TiO_2_-coated FTO slides were rinsed, dried, and calcined at 550 °C for 1 h in air.

### 2.3. Fabrication of TiO_2_-RuO_x_ Cathodes

The as-prepared TiO_2_ NAs/FTO were immersed in RuCl_3_ solutions of varying concentrations (0.5, 1.0, and 1.5 mM) for 30 min, followed by drying under N_2_ flow and calcinatiion at 500 °C for another 1 h in air. The resulting samples were denoted as TiO_2_/Ru-0.5, TiO_2_/Ru-1.0, and TiO_2_/Ru-1.5, respectively.

### 2.4. Characterization

The as-prepared morphology of the cathodes was characterized by scanning electron microscopy (SEM, -JSM-7900F, JEOL, Akishima, Japan) and transmission electron microscopy (TEM, JEM-1400plus, JEOL, Akishima, Japan). The crystal structures of samples were examined using X-ray diffraction (XRD, Smart Lab III, Rigaku, Tokyo, Japan). The absorbance spectra of the ClO_2_ solutions were measured using a UV–vis spectrophotometer (UV-9000, Metash Instrument Co., Ltd., Shanghai, China). The surface chemical states of cathodes were analyzed using X-ray photoelectron spectrometry (XPS, ESCALAB 250XI, ThermoFisher Scientific, East Grinstead, UK).

### 2.5. Generation and Quantification of ClO_2_

ClO_2_ was generated in an H-type electrochemical cell with a CHI660E potentiostat. A 2 × 2 cm^2^ Pt mesh and an Ag/AgCl electrode served as counter and reference electrodes, respectively. The electrolyte solution consisted of H_2_SO_4_ and 1 M NaClO_3_. ClO_2_ was generated at constant cathodic potentials, and its concentration in the electrolyte solution was quantified by UV–vis spectroscopy based on the characteristic absorption at 360 nm.

The rotating ring-disk electrode (RRDE) tests were performed under O_2_ conditions with a rotation speed of 1600 rpm and ring voltage of 0.93 V vs. Ag/AgCl.

### 2.6. Antimicrobial Activity Test

To evaluate the antibacterial activity of gaseous ClO_2_, the ClO_2_ produced during 30 min of electrolysis was subsequently transferred into a gas collection chamber using N_2_ as the carrier gas (0.8 L min/L). Gram-negative *Escherichia coli* (*E. coli*) and Gram-positive *Staphylococcus aureus* (*S. aureus*) were employed as model microorganisms. Agar plates were prepared by pouring 20 mL of sterilized beef extract–peptone agar medium into Petri dishes, followed by solidification for 10 min. Then, 100 μL of freshly prepared bacterial suspensions (10^8^ CFU/mL for both *S. aureus* and *E. coli*) were uniformly inoculated onto the agar plates. The agar plates in the experimental group were placed and exposed in the ClO_2_ chamber for 1 h, while those in the control group were not. Then all plates were incubated at 37 °C in air for 24 h, followed by counting the bacterial colonies.

The antibacterial efficacy (*AE*%) was calculated using the following equation:

$$\mathrm{AE}\%\;=\;\frac{N_{E}}{N_{C}}\times 100\%$$

where *N*_E_ and *N*_C_ are the number of bacteria colonies surviving in the experimental group and the control group, respectively.

### 2.7. Food Preservation

Fresh longans were rinsed with sterile running water and dried under sterile conditions, grouped into sets of seven, and placed in preservation chambers. Electrochemically generated ClO_2_ gas was introduced into the chambers of the experimental group, while the control group was not exposed to ClO_2_. All the longans were incubated at room temperature for 8 days for quality determination.

The longans were weighed via an analytical balance on days 0, 2, 4, 6 and 8. The weight loss was calculated via following formula:

$$\mathrm{Weight}\;\mathrm{loss}\;=\;\frac{w_{Day0}-w_{\mathrm{Final}}}{w_{\mathrm{Day}0}}\times 100\%$$

where *w*_Day0_ is the original weight of longans, and *w*_Final_ is the weight of longans at the corresponding day.

The hardness of the longans was determined using a fruit hardness tester (Lexmark Edelbrock Co., Ltd., Berkshire, UK) with a 3.5 mm cylindrical probe. The probe was inserted into the equatorial region of the longan, and the maximum penetration force was recorded as the hardness value (the data were averaged).

## 3. Results and Discussion

### 3.1. Morphology and Structure of TiO_2_ NAs

**Figure 1:**
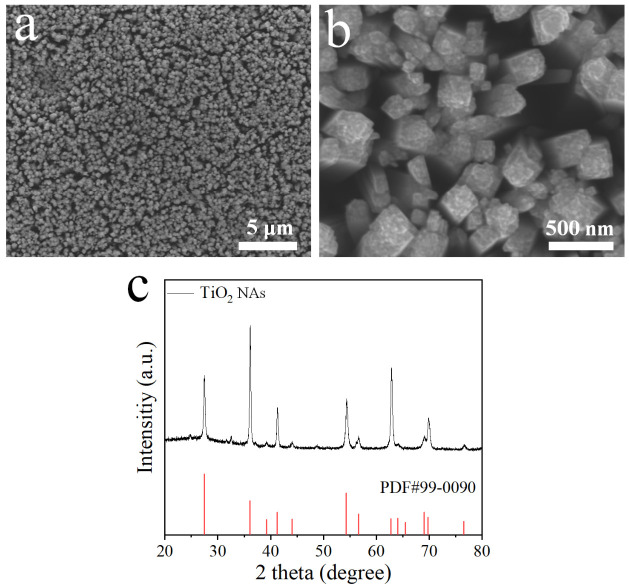
(**a**,**b**) SEM images and (**c**) XRD pattern of TiO_2_ NAs.

The morphology and crystal structure of the TiO_2_ nanorod arrays (NAs) synthesized on FTO substrates were investigated by SEM and XRD, respectively. As shown in Figure 1a the TiO_2_ nanorods are vertically and uniformly grown across the FTO surface, forming a continuous and well-adhered array. Figure 1b reveals the tetragonal faceted-nanorod structure of TiO_2_ NAs and open inter-rod spaces that may create an extensive electrode–electrolyte interface and facilitate mass transport [29]. The XRD pattern (Figure 1c) shows that all diffraction peaks can be indexed to rutile TiO_2_ (PDF#99-0090), indicating high crystallinity and phase purity for TiO_2_ NAs after annealing in air at 550 °C. Consequently, this vertically aligned rutile nanorod architecture promises direct electron transport pathways and enhanced structural stability in strong acidic electrolytes, which are advantageous for efficient electrochemical generation of ClO_2_ [30,31].

### 3.2. Electrochemical Generation of ClO_2_

**Figure 2:**
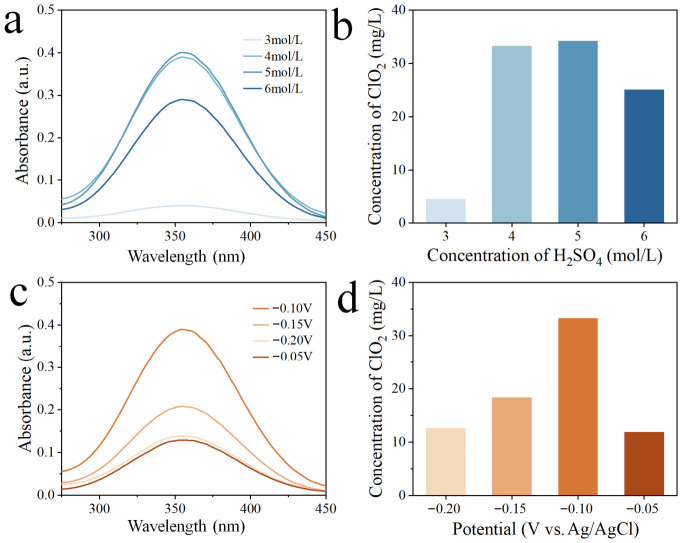
(**a**) UV–vis absorption spectra and (**b**) corresponding concentrations of electrochemically generated ClO_2_ with varying H_2_SO_4_ concentrations in electrolyte solutions. (**c**) UV–vis absorption spectra and (**d**) corresponding concentrations of generated ClO_2_ under varying cathodic potentials.

In order to optimize the efficiency of ClO_2_ generation, the effects of H_2_SO_4_ concentration and the applied potential were first systematically investigated. As shown in Figure 2a,c, after cathodic electrolysis, the UV–vis absorption spectra of the electrolyte solutions exhibit a characteristic absorption at approximately 360 nm, confirming the successful formation of ClO_2_ [32]. The corresponding ClO_2_ concentrations (Figure 2b,d) were quantified based on the established standard curve (Figure S1). The concentration of H_2_SO_4_ is critical in ClO_2_ production systems, as it directly influences both the reaction pathway and product stability [33,34]. Accordingly, the effect of H_2_SO_4_ concentration was evaluated by chronoamperometry at a fixed potential of −0.10 V vs. Ag/AgCl, with the NaClO_3_ concentration fixed at 1 M. As illustrated in Figure 2a,b, the ClO_2_ yield increases markedly as the H_2_SO_4_ concentration is increased from 3 M to 5 M, followed by a decline at 6 M. The influence of applied potential was then examined at a fixed electrolyte composition of 4 M H_2_SO_4_ and 1 M NaClO_3_. Chronoamperometry tests were conducted at potentials ranging from −0.05 V to −0.20 V vs. Ag/AgCl. As shown in Figure 2c,d, the concentration of generated ClO_2_ initially increases with increasing cathodic potentials and reaches a maximum at −0.10 V, after which it decreases at more negative potentials (−0.2 V vs. Ag/AgCl). This volcano-type dependence indicates that mild potential limits ClO_2_ production due to insufficient driving force, whereas excessive overpotential likely promotes competing side reactions, particularly H_2_ evolution, thereby reducing the ClO_2_ yield. Overall, 4 M H_2_SO_4_ and −0.10 V vs. Ag/AgCl were selected as the optimal operating potential for ClO_2_ generation in our work.

**Figure 3:**
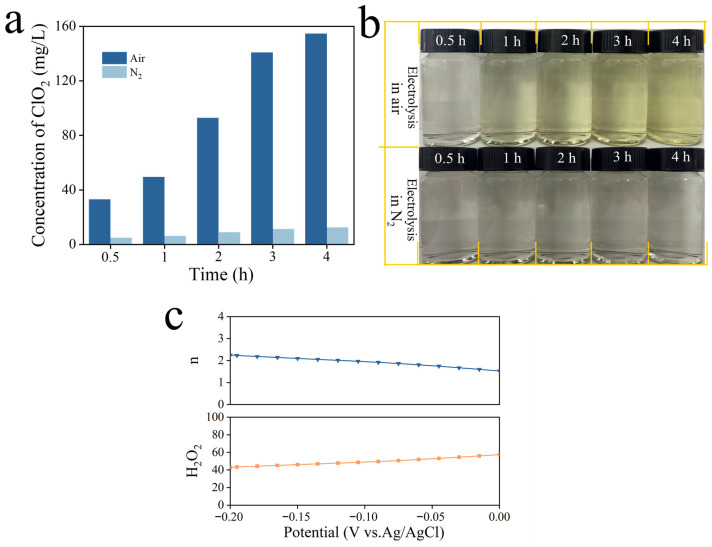
(**a**) The concentrations of the generated ClO_2_ and (**b**) corresponding digital photos of the electrolyte solutions during 4 h electrolysis by the pristine TiO_2_ NA cathode under air and N_2_ atmospheres. (**c**) Electron transfer number (n) and H_2_O_2_% of TiO_2_ NAs at −0.1 V vs. Ag/AgCl in O_2_^−^ saturated electrolyte solution of 5 M H_2_SO_4_ in the absence of NaClO_3_.

To elucidate the generation mechanism of ClO_2_ by the pristine TiO_2_ NA cathode, the influence of dissolved O_2_ was investigated by comparing ClO_2_ production under air and N_2_ atmospheres. As shown in Figure 3a, under air conditions, the concentration of ClO_2_ increases continuously with electrolysis time, reaching ~ 150 mg L^−1^ after 4 h. In sharp contrast, when kept purging with N_2_, the ClO_2_ concentration remained extremely low throughout the 4 h electrolysis. Additionally, the i-t curves (Figure S2) reveal that the cathodic current density under N_2_ is remarkably lower than that under air, indicating that dissolved O_2_ acts as the dominant electron acceptor during the electrolysis process and governs the ClO_2_ generation pathway. The comparison of the electrolyte solutions (Figure 3b) is consistent with this conclusion, as a progressively deepening yellow color of ClO_2_ is observed under air conditions, whereas solutions electrolyzed under N_2_ remain nearly colorless. Previous studies have reported that TiO_2_ is capable of catalyzing the 2-e^−^ reduction of O_2_ to H_2_O_2_ [35,36], and thus it is reasonable to propose that ClO_2_ formation proceeds via the reaction between in situ generated H_2_O_2_ and ClO_3_^−^, instead of direct reduction of ClO_3_^−^ to ClO_2_.

We further evaluated 2e^−^ ORR performance of the TiO_2_ NA cathode under O_2_ atmosphere by employing rotating ring-disk electrode (RRDE) measurements in the absence of NaClO_3_. As shown in Figure 3c, the electron transfer number (n) was determined to be close to ~2.0 over the investigated potential range, accompanied by a considerable H_2_O_2_ selectivity of approximately 40–60%. These results clearly demonstrate that the oxygen reduction reaction on TiO_2_ NAs predominantly follows a two-electron pathway, leading to the in situ generation of H_2_O_2_. This is attractive from both an environmental and practical perspective, since most commercial ClO_2_ production systems involve the use and storage of H_2_O_2_, thereby leading to operational costs and potential safety risk. This O_2_-induced electrochemical system avoids this issue.

Based on these findings, it is reasonable to propose that ClO_2_ formation proceeds via an indirect pathway, in which electrochemically generated H_2_O_2_ reacts with ClO_3_^−^ in the electrolyte, rather than through direct electroreduction of ClO_3_^−^. This mechanism not only explains the strong dependence on dissolved O_2_, but also highlights the advantage of this system in avoiding the external addition and storage of H_2_O_2_, thereby improving operational safety and practicality.

### 3.3. TiO_2_/RuO_x_ Composite Cathodes

**Figure 4:**
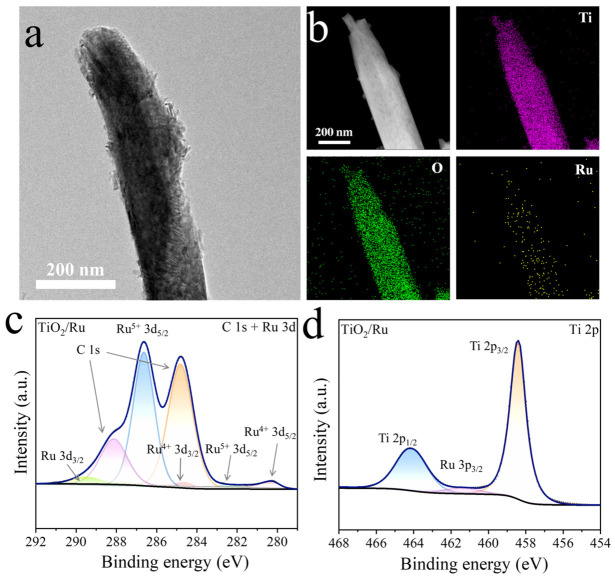
(**a**) The TEM image and (**b**) energy-dispersive X-ray (EDX) mapping for different elements (O, Ti, O and Ru) for an individual TiO_2_ nanorod decorated with RuO_x_ nanoparticles. High-resolution XPS spectrum of (**c**) Ru 3d (overlapping with C 1s) and (**d**) Ti 2p for TiO_2_/RuO_x_.

To further enhance the ClO_2_ production efficiency, RuO_x_ nanoparticles were loaded onto the TiO_2_ NAs via a Ru^3+^ solution impregnation–calcination process. As shown in Figure 4a, RuO_2_ nanoparticles uniformly grew on the surface of the TiO_2_ nanorod.

Elemental mapping results (Figure 4b) shows that Ru is uniformly dispersed along the TiO_2_ nanorod, suggesting that RuO_x_ particles are well distributed rather than aggregated. Figure S4 presents the energy-dispersive spectrum of TiO_2_/RuO_x_ scratched from the FTO glass substrate. The atomic ratio of O:Ti:Ru is 60.41: 39.47: 0.12, corresponding to a Ru content of ~0.12 at%, confirming that Ru is present in a relatively low content. In addition, the surface elemental composition and chemical states of the resulting TiO_2_/RuO_x_ composites were investigated by XPS analysis. As shown in Figure 4c, the high-resolution Ru 3d XPS spectrum exhibits two characteristic peaks located at 280.3 eV and 284.6 eV, which can be assigned to the Ru^4+^ 3d_5/2_ and Ru^4+^ 3d_3/2_ in RuO_2_ species, respectively. In addition, Ru^5+^ peaks appearing at 282.8 eV and 286.9 eV further confirm the presence of oxidized Ru species. The peak located at 289.49 eV can be assigned to Ru 3d_3/2_ [37,38]. The C 1s peaks are revealed at 284.8 eV and 288.12 eV, originating from adventitious carbon contamination [39]. The Ti 2p spectrum (Figure 4d) displays two peaks at 458.4 eV and 464. 1 eV, corresponding to the Ti 2p_3/2_ and Ti 2p_1/2_ levels of Ti^4+^, respectively [40,41]. Notably, a weak peak around 461.4 eV can be assigned to Ru 3p_3/2_, further confirming the successful incorporation of Ru^4+^ species [42].

**Figure 5:**
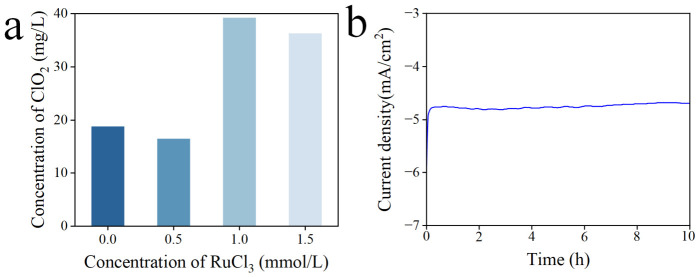
(**a**) Concentration of ClO_2_ generated using TiO_2_/RuO_x_ cathodes prepared with different RuCl_3_ precursor concentrations. (**b**) Long-term i-t curve of TiO_2_/Ru-1.0 in the electrolyte solution containing 1 M NaClO_3_ and 5 M H_2_SO_4_ at a constant potential of −0.1 V vs. Ag/AgCl.

In addition, we optimized the concentration of the RuCl_3_ precursor solution. Figure 5a shows that the highest ClO_2_ yield is achieved using a 1.0 mmol/L RuCl_3_ precursor, directly correlating with the highest absorbance intensity among those samples (Figure S3). Moreover, to examine the long-term stability of the TiO_2_/RuO_x_ cathode, we applied a continuous electrolysis to the TiO_2_/Ru-1.0 cathode for 10 h at a constant potential of −0.1 V (vs. Ag/AgCl) in an electrolyte of 1 M NaClO_3_ and 5 M H_2_SO_4_. Figure 5b exhibits that the current density remains remarkably steady with negligible loss, demonstrating the high stability of the TiO_2_/RuO_x_ cathode under the operational condition and confirming its potential for practical applications.

### 3.4. Antibacterial and Fruit Preservation Applications

**Figure 6:**
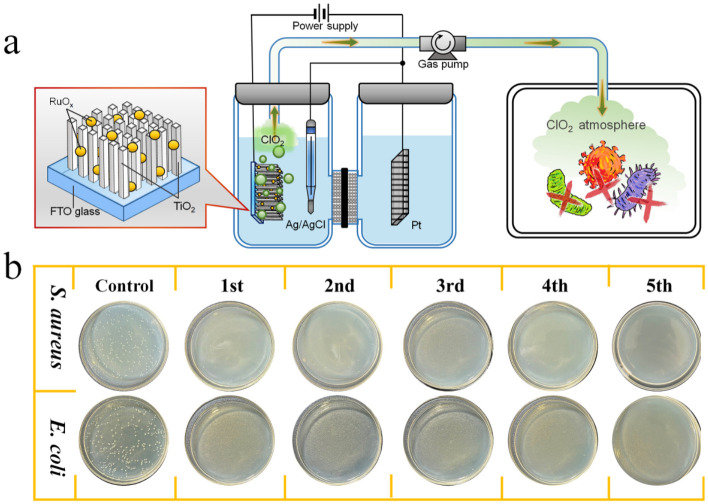
(**a**) Schematic diagram of the gaseous ClO_2_ generation system based on the TiO_2_/RuO_x_ cathodes. (**b**) Evaluation of the antibacterial effect of the emulsion gel by colony counting method.

To evaluate the feasibility of the TiO_2_/RuO_x_ cathodes for practical sterilization, a gaseous ClO_2_ delivery system was constructed. As illustrated in Figure 6a, ClO_2_ was first generated in the electrolyte solution via cathodic chronoamperometry for 30 min (using TiO_2_/Ru-1.0 as the working electrode). To facilitate the transformation of ClO_2_ to the gas phase, meanwhile, N_2_ was subsequently introduced as a carrier gas at a controlled flow rate (0.8 L/min), effectively delivering ClO_2_ into a gas collection chamber. The steady-state concentration in the sterilization environment was monitored as ~7.5 ppm with a ClO_2_ gas detector. The antibacterial performance of the generated ClO_2_ gas was evaluated against *E. coli* and *S. aureus* using the colony counting method. As shown in Figure 6b, after exposure to the ClO_2_ atmosphere for 1 h, the treated agar plates exhibited a complete absence of visible colonies for both *E. coli* and *S. aureus* colonies, achieving > 99% antibacterial efficacies. In contrast, the control group exposed to air exhibited significantly higher bacterial survival rates. Furthermore, the long-term practical utility was assessed through cyclic antibacterial tests. Even after five consecutive cycles, the antibacterial efficacy remained consistently above 95%. These results indicate that the TiO_2_/RuO_x_ cathode system combines efficient catalytic activity towards ClO_2_ generation with good stability, highlighting its potential for long-term disinfection applications.

**Figure 7:**
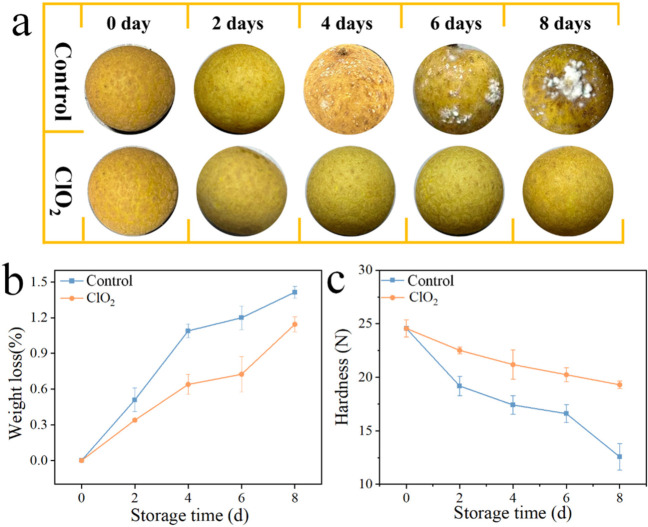
(**a**) Visual appearance, (**b**) weight loss and (**c**) hardness comparison of longans between experimental (exposed to ClO_2_) and control groups (exposed to air) over storage time.

To validate the practical applicability of our TiO_2_ NA gaseous ClO_2_ preservation system, longan was selected as the model fruit due to its high perishability, thin pericarp, and susceptibility to microbial spoilage under ambient conditions. Figure 7a displays the visual appearance of longan samples from both experimental group (placed in the ClO_2_ chamber) and control group (exposed to air) at different durations. It is clearly observed that longans in the control group are covered with white mold and emitting rotten odor, whereas the experimental group shows remarkable freshness retention. This demonstrates that bacteria growth has been effectively inhibited by the ClO_2_ atmosphere during the 8-day storage period. Additionally, to further quantify the preservative effects of the electrochemically generated ClO_2_ atmosphere, the physical quality parameters, including weight loss and fruit hardness, were monitored over the 8-day storage period. As shown in Figure 7b, the experimental group exhibited significantly lower weight loss rate than the control group. This can be attributed to the fact that ClO_2_ effectively inhibits the microbial metabolism on the longan surface. Moreover, the experimental group maintained significantly higher hardness compared to the control group, as shown in Figure 7c, indicating the effective structural preservation of longan tissues. All in all, these results verify that our system provides an effective source of gaseous ClO_2_ capable of extending the shelf life of fruits by reducing microbial-induced decay and maintaining texture of fruits.

## 4. Conclusions

In summary, this study successfully developed a high-efficiency electrochemical system for the in situ generation of gaseous ClO_2_ using TiO_2_ NA cathodes. To achieve the optimal ClO_2_ yield, the electrolysis was conducted at a cathodic potential of −0.10 V vs. Ag/AgCl in an electrolyte solution of 1 M NaClO_3_ with 5 M H_2_SO_4_ utilizing a TiO_2_ NA cathode modified with RuO_x_ nanoparticles via a 1.0 mM RuCl_3_ precursor solution. Mechanistic investigations reveal that the generation of ClO_2_ was governed by an O_2_-induced pathway, where dissolved O_2_ was first reduced to H_2_O_2_ at the electrode surface and the in situ generated H_2_O_2_ reacted with ClO_3_^−^ to form ClO_2_. This offers a safer and more cost-effective alternative to traditional ClO_2_ production systems requiring the external storage of H_2_O_2_. Practical application of the generated gaseous ClO_2_ achieved >99% antibacterial efficacy against *E. coli* and *S. aureus*, with performance remaining above 95% even after five disinfection cycles. Furthermore, the system proved highly effective for fruit preservation, significantly reducing weight loss and maintaining fruit hardness in longans during an 8-day storage period. These findings highlight the TiO_2_ NA system as a stable, efficient, and versatile platform for on-demand disinfection and advanced food preservation technologies.

## Data Availability

The original contributions presented in this study are included in the article/Supplementary Material. Further inquiries can be directed to the corresponding author.

## Supplementary Materials

The following supporting information can be downloaded at https://www.mdpi.com/article/10.3390/ma19091674/s1, Figure S1: UV-vis absorbance spectra of aqueous ClO_2_ solutions with varying concentrations; Figure S2: The i-t curves of TiO_2_ NAs recorded in 1 M NaClO_3_ with 5 M H_2_SO_4_ at −0.1 V vs. Ag/AgCl under air and N_2_ atmospheres; Figure S3: UV-vis absorbance spectra of aqueous ClO_2_ generated using TiO_2_/RuO_x_ cathodes prepared with different RuCl_3_ precursor concentrations; Figure S4: The energy dispersive spectrum TiO_2_/RuO_x_ scratched from the FTO glass. The Cu signal originates from the copper grid used as the specimen holder. The atomic ratio of O:Ti:Ru is 60.41:39.47:0.12.
