# Supplementary material for: An Electrochemical System for Gaseous ClO2 Generation Using TiO2 Nanorod Array Cathodes Toward Fruit Preservation
Source: Materials (Basel). 2026 Apr 22;19(9):1674. doi: 10.3390/ma19091674 (PMC13165234; doi:10.3390/ma19091674)
Supplement: Supplementary file 1 [file materials-19-01674-s001.zip › materials-4223678-supplementary.pdf]

An Electrochemical System for Gaseous ClO_2 Generation Using TiO_2 Nanorod Array cathodes toward Food Preservation

Luyi Pang,[a] Junyuan Jiang, [a], Rengui Guan, [a] Yanyang Han, [a] Shanshan Liu, [a] Shasha Jiang, [a] Wei Cui[a]* and Tao He [a]

a: College of Chemistry and Chemical Engineering, Yantai University, No. 30 Qingquan Road, 264005, P.R. China

*Corresponding Author

E-mail addresses: wcui@ytu.edu.cn (Wei Cui)

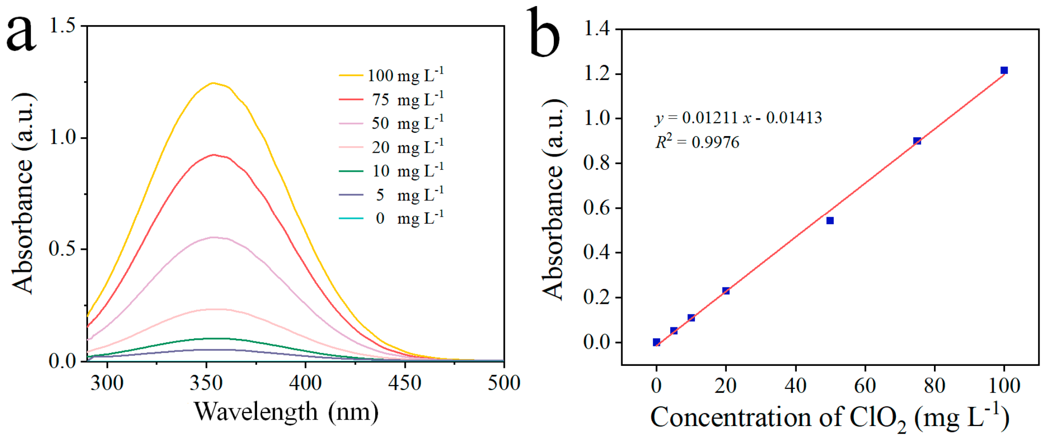

Figure S1 UV-vis absorbance spectra of aqueous ClO_2 solutions with varying concentrations, (b) standard curve of absorbance at 360 nm against ClO_2 concentration.

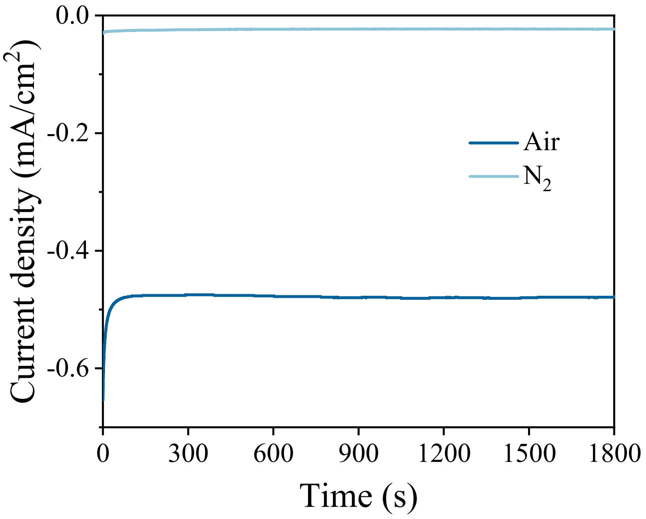

Figure S2 The i-t curves of TiO_2 NAs recorded in 1 M $NaClO_3$ with 5 M H_2SO_4 at -0.1 V vs. Ag/AgCl under air and N_2 atmospheres.

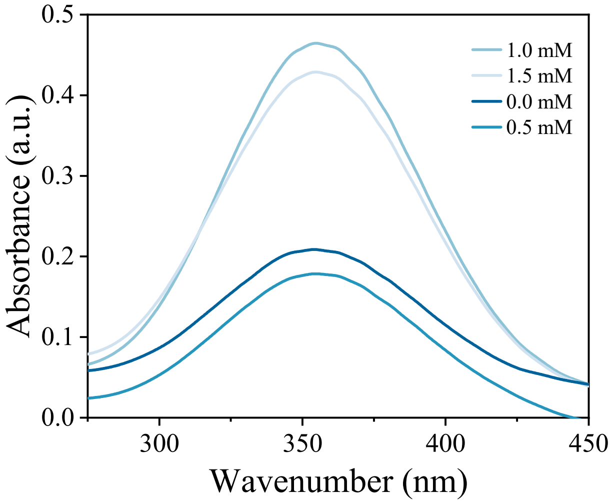

Figure S3 UV-vis absorbance spectra of aqueous ClO_2 generated using TiO_2/RuO_x cathodes prepared with different $RuCl_3$ precursor concentrations.

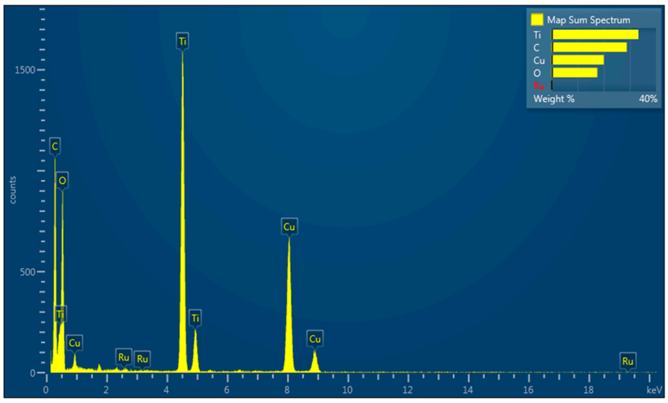

Figure S4 The energy dispersive spectrum TiO_2/RuO_x scratched from the FTO glass. The Cu signal originates from the copper grid used as the specimen holder. The atomic ratio of O:Ti:Ru is 60.41:39.47:0.12.
